# Serum Iron Markers in Patients With Chronic Hepatitis C Infection

**DOI:** 10.5812/hepatmon.13136

**Published:** 2013-10-05

**Authors:** Codruta Vagu, Camelia Sultana, Simona Ruta

**Affiliations:** 1 Discipline of Biochemistry, Carol Davila University of Medicine and Pharmacy, Bucharest, Romania; 2 Department of Virology, Carol Davila University of Medicine and Pharmacy, Bucharest, Romania; 3 Emergent Diseases Department, Stefan S. Nicolau Institute of Virology, Bucharest, Romania

**Keywords:** Chronic Hepatitis C, Ferritin, Transferrin, Liver Cirrhosis

## Abstract

**Background:**

Patients with chronic hepatitis C (CHC) often have elevated serum iron markers, which may worsen liver injury.

**Objectives:**

The aim of this study was to investigate the possible correlations between iron metabolism serum markers, HCV viral load, and liver disease severity in treatment-naive patients with chronic hepatitis C infection.

**Patients and Methods:**

Eighty five patients with untreated hepatitis C chronic infection were investigated.

**Results:**

Twenty one patients (24.7%) had elevated serum iron levels, and 29 subjects (34.1%) had severe liver fibrosis. Significantly elevated levels of serum iron (P < 0.05) and ferritin (P < 0.001), associated with lower levels of TIBC (P < 0.05) were detected in patients with severe fibrosis compared to no/mild fibrosis. Severe necroinflammatory activity was also significantly correlated with serum iron (P < 0.001), TIBC (P < 0.05), and ferritin levels (P < 0.001). Using multiple linear regression analysis, serum levels of ferritin and transferrin were the independent variables selected as being good predictors for advanced fibrosis and severe necroinflammatory activity. No significant correlations were detected between HCV viral load and iron markers.

**Conclusions:**

This study revealed that serum iron markers (especially ferritin and transferrin) might be used as surrogate markers for both liver fibrosis and necroinflammatory activity.Patients with chronic hepatitis C (CHC) often have elevated serum iron markers, which may worsen liver injury.

## 1. Background

Hepatitis C virus (HCV) infection is one of the major global public health concerns. More than 170 million people have positive results for hepatitis C antibody, with an estimated worldwide prevalence of 3% (1, 2).

Chronic Hepatitis C (CHC) progression to cirrhosis within 2-3 decades is reported in up to 20% of cases; a quarter of these would develop decompensated liver disease, hepatocellular carcinoma and would need liver transplantation (3). The outcome of an HCV infection depends on viral factors (baseline viral load and genotype), host genetic background and comorbidities.

The liver is the main iron storage organ, a third of the body’s total iron is deposited in hepatocytes, in the portal tracts, sinusoidal mesenchymal cells, and reticuloendothelial cells (4, 5). It also plays a fundamental role in iron metabolism, as both transferrin (the main transporting protein) and ferritin (the major storage protein) are synthesized here. Several studies have reported that elevated serum iron markers (ferritin, iron, and transferrin saturation) and iron accumulation within the liver occur frequently in patients with CHC infection and may worsen liver injury (6-8). It was reported that stages III and IV of fibrosis may go undetected, with minimal or no clinical symptoms and signs, so that, detecting severe fibrosis or cirrhosis in CHC is of prognostic importance (9), because the risk of liver decompensation is very high in patients with advanced liver fibrosis (2).

## 2. Objectives

The aim of this study was to investigate the possible correlations between iron metabolism serum markers, HCV viral load, and liver disease severity in treatment-naive patients with chronic hepatitis C infection.

## 3. Patients and Methods

This analytical retrospective study was conducted during June 2012 and January 2013, in a referral center in Bucharest where 117 HCV-infected untreated patients were admitted mainly for evaluation of HCV infection. From them 85 patients were included in the study based on the presence of serum HCV antibody for at least six months using an electrochemiluminescence assay (Roche Elecsys Anti-HCV assay). The exclusion criteria were: patients with positive results for screening tests for other viral hepatitis infections (anti-HAV IgM, HBsAg, anti-HBc IgM) or HIV infection, hepatic cancer or other types of malignancies, hematological or non-viral chronic liver diseases, and patients previously treated for HCV infection.

No formal sample size calculation was performed, all the study patients had to have active viral replication confirmed by detecting HCV-RNA, using real-time polymerase chain reaction assay (COBAS Taqman HCV Test, Roche - range of quantification: 43 IU/mL to 6.9 x 107 IU/mL, limit of detection -18 IU/mL, with a positivity rate greater than 95%).

Informed consent was obtained from all patients, patient privacy was respected over the testing and analyzing data and the study was approved by the Bioethics Committee of the Stefan S. Nicolau Institute of Virology. All data exchanged and stated by the patients remained confidential.

HCV genotyping was performed using a reverse hybridization assay Versant HCV genotype 2.0 assay, Bayer Healthcare Division, according to the manufacturer instructions.

Liver fibrosis and necroinflammatory activity grade was appreciated using biochemical composite scores – FibroTest (FT) and ActiTest (AT) - panels of biochemical markers with values from 0 to 1, with conversion in the METAVIR system from F0 to F4 for liver fibrosis and A0 to A3 for the necroinflammatory activity. The FT identifies about 70% of patients with histological signs of moderate to severe fibrosis, and about 90% of patients with cirrhosis. The negative predictive value for excluding significant necroinflammatory activity of AT is 85% (10).

Serum alanine aminotransferase (ALT), aspartate aminotransferase (AST), serum iron, and unsaturated iron-binding capacity (UIBC) were monitored with automated analyzer (Roche Modular P chemistry analyzer). Ferritin was determined by an electrochemiluminescence assay (Roche Elecsys-e170) and serum transferrin by immunoturbidimetric method (Roche Modular on ECLIA analyzer). Total iron binding capacity (TIBC) was calculated as the sum of serum iron and UIBC values.

### 3.1. Statistical Methods

Descriptive statistic was applied to assume data distribution, mean and standard deviation; for mean comparison and their statistical significance was used t-test, parametric and nonparametric tests; for association between baseline predictors of chronic hepatitis C with serum indicators a multiple linear regression analysis was applied.

Data was gathered in excel spreadsheet and statistics was performed in IBM SPSS v.20. A P value < 0.05 was considered statistically significant.

## 4. Results

### 4.1. Patients’ Characteristics

The mean age of patients was 49.5 ±12.5 years (ranging from 20 to 72 years), and more than a half were males (male/female index=1.5). Only 29 of 85 subjects (34.1%) had severe liver fibrosis and 23 subjects (27.1%) had severe necroinflammatory activity. Seventeen patients (20%) had normal serum ALT level, while another twenty one (24.7%) had high serum ALT levels (two times higher than the upper limit of normal- ULN which is 40 U/L). Viral load was high (>5,77 log10 UI/mL) in 56 patients (65.9%), and HCV genotype 1 was the most prevalent. HCV subtype 1b was identified in 97.6% of the cases, 1a in 1.2%, and mixed genotypes in 1.2% patients.

Twenty one patients (24.7%) had high serum iron levels, 61 (71.7%) presented normal levels related to age and sex (ranging between 59 to 158 μg/dL), and the rest (3 patients, 3.5%) had levels below 59 μg/dL. Only four patients (4.7%) had serum ferritin exceeding 400 ng/L, 73 (85.9%) had normal values (between 30-400 ng/L), and 8 (9.4%) patients had values under 30 ng/L. Sixteen patients (18.8%) had serum transferrin values higher than 3.6 g/L, while the rest 69 (81.1%) had normal levels (ranging between: 2-3.6 g/L). Thirty patients (35.3%) had TIBC values less than 228 μg/dL without having anemia, while 55 (64.7%) of them presented normal values (between 228-428 μg/dL) (Table 1). 

**Table 1.** Baseline Characteristics of the Patients

Patients characteristics	Value, No. (%)
**Gender**	
Male	51 (60)
Female	34 (40)
**Age,y ^**a**^**	49.5 ± 12.5
**Genotype**	
1b	83 (97.6)
1a	1 (1.2)
Mixed	1 (1.2)
**ALT, IU/L ^**a**^**	76.1 ± 73.7
Normal < 40	17 (20)
Between 40-80	47 (55.3)
> 2 X ULN ^b^	21 (24.7)
**AST, IU/L ^**a**^**	59.04 ± 56.8
Normal < 40	36 (42.4)
Between 40-80	38 (44.7)
2 X ULN ^b^	11(12.9)
**Serrum Ferritin, ng/L ^**a**^**	159.7 ± 118.07
< 30	8 (9.4)
Normal (30-400)	73 (85.9)
> 400	4 (4.7)
**Serum iron, μg/dL ^**a**^**	126.05 ± 47.2
< 59	3 (3.5)
Normal (59-158)	61 (71.7)
> 158 μg/dL	21 (24.7)
**Transferrin, g/L ^**a**^**	3.2 ± 0.4
Normal (2-3.6)	69 (81.1)
> 3.6	16 (18.8)
**Mean TIBC ^**a**^**	251.5 ± 68.9
Normal (228-428)	55 (64.7)
< 228 μg/dL	30 (35.3)
**Mean viral load, log_10_ IU/mL**	6.18 log_10_ (6.07 log_10_-6,26 log_10_)
HCV RNA < 5.77	29 (34.1)
HCV RNA > 5.77	56 (65.9)
**Stage of liver fibrosis**	
Mild/absent (F0/F1)	36 (42.4)
Moderate (F2)	20 (23.5)
Severe (F3/F4)	29 (34.1)
**Grade of necroinflammatory activity**	
Mild/absent (A0/A1)	47 (55.3)
Moderate (A2)	15 (17.6)
Severe (A3)	23 (27.1)

^a^ Mean ± SD

^b^ Upper limit normal

There was no correlation between the level of viral replication and the values of iron metabolism markers.

### 4.2. Correlation between Iron Metabolism Markers and the Severity of Liver Disease

34.1% of the patients had severe fibrosis (as defined by F3/4 FibroTest scores); these patients tend to be older (the mean age 56.25±7.55 years vs. 46.19±13.18 years; P < 0.001), and had higher ALT levels (the mean ALT value 110.10 ± 107.91 IU/L vs. 59.46 ± 41.10 IU/L; P < 0.001) than patients with no/mild fibrosis (Table 2). We observed significantly elevated levels of serum iron (P < 0.05) and ferritin (P < 0.001), associated with lower levels of TIBC (P < 0.05) in patients with severe fibrosis compared to those with no/mild fibrosis stages. 

**Table 2.** Association between Liver Fibrosis, Necroinflammatory Activity and Serum Iron Markers

Variable	Total, n = 85	No/Moderate fibrosis (F0/F1/F2), n = 56 (65.9%)	Severe fibrosis (F3/F4), n = 29 (34.1%)	P value	Absent/moderate necroinflammatory activity (A0/A1/A2), n = 62 (72.9%)	Severe necroinflammatory activity (A3), n = 23 (27.1%)	P value
**Age, y**	49.5 ± 12.5	46.19 ± 13.18	56.25 ± 7.55	< 0.001	48.43 ± 12.83	52.59 ± 11.28	> 0.999
**AST ** ^**a**^ **(IU/L) ^ b^**	59.05 ± 56.81	45.28 ± 36.81	87.07 ± 77.56	< 0.001	39.71 ± 17.34	114.40 ± 87.70	< 0.001
**ALT ** ^**a**^ **(IU/L) ^ b^**	75.14 ± 73.77	59.46 ± 41.10	110.10 ± 107.91	< 0.001	49.51 ± 20.80	152.40 ± 110.79	< 0.001
**SerumIron, μg/dL ^b^**	126.05 ± 47.22	115.19 ± 35.30	148.17 ± 59.97	< 0.05	114.25 ± 34.58	159.87 ± 61.43	< 0.001
**TIBC** ^**a**^ **, μg/dL ^ b^**	251.55 ± 68.92	263.46 ± 61.29	227.30 ± 77.97	< 0.05	262.21 ± 64.54	221.02 ± 73.40	< 0.05
**Ferritin** ^**a**^ **, ng/mL ^ b^**	159.68 ± 118.07	125.87 ± 88.43	228.53 ± 140.79	< 0.001	133.48 ± 103.65	234.75 ± 126.80	< 0.001
**Transferrin, g/L ^ b^**	3.26 ± 0.41	3.23 ± 0.38	3.30 ± 0.47	> 0.999	3.21 ± 0.42	3.38 ± 0.35	> 0.999
**Viral load ^ b^**	1.5×106 ± 1.4×106	1.6×106 ± 1.6×106	1.3×106 ± 1 ×106	> 0.999	1.6 ×106 ± 1.6 ×106	1.4×106 ± 0.7 ×106	> 0.999

^a^ Abbreviations: ALT, Serum alanine aminotransferase; AST, Serum aspartate aminotransferase; TIBC, Total iron binding capacity

^b^ Mean ± SD

Twenty three patients (27.1%) had severe necroinflammatory activity (as defined by A3 ActiTest scores), high viral load (mean value: 1. 4x106 ± 0.7 × 106) and their mean age was higher (52.59 ± 11.28 years vs. 48.43 ± 12.83 years; P > 0.999) than patients with absent/moderate necroinflammatory activity. We noticed that severe necroinflammatory activity was directly correlated with three serum iron markers (serum iron level P < 0.001; TIBC P < 0.05; ferritin P < 0.001) (Table 2). 

High iron and ferritin levels were accompanied by elevations in serum hepatic aminotransferase levels in patients with severe fibrosis and also with severe necroinflammatory activity, although these correlations did not reach statistical significance.

Using multiple linear regression analysis routine in SPSS software, serum levels of ferritin and transferrin and also serum iron in one of the linear regression models, were the independent variables automatically selected as good predictors for advanced fibrosis and severe necroinflammatory activity. After performing partial correlation routine to avoid confounders influence, we excluded values of transaminases (it is known to have high level in severe fibrosis and necroinflammatory activity) and age from linear regression analyze. Also viral load and gender did not show statistical significance. The predictive model using serum levels of transferrin and ferritin had higher statistical significance for severe fibrosis (F(2.28) = 366.317, P < 0.001, R^2^adj = 0.963) vs. absent/moderate fibrosis (F0/F1/F2) (F(2,57) = 91.141, P < 0.001, R^2^ adj = 0.760), while higher statistical significance for severe necroinflammatory activity (A3) (F(2.22) 591.976, P < 0.001, R^2^ adj = 0.982) vs. absent/moderate necroinflammatory activity (A0/A1/A2) (F(1.63) = 96.668, P < 0.001 R^2^ adj = 0.603) was obtained using transferrin and serum iron levels.

## 5. Discussion

Iron homeostasis is critical for human organism and the pathogenesis of iron accumulation in CHC is not yet completely understood. The present study indicates that even though most naive CHC patients have blood serum iron markers within the normal ranges; elevated serum levels of iron, ferritin, and transferrin can represent early markers for the severity of liver disease, related both to the degree of liver fibrosis and to the necroinflammatory activity. Increased levels of ferritin and serum iron levels and the decreased levels of TIBC were correlated with progressive hepatic parenchymal disease. A possible explanation for these elevations is that a necroinflammatory hepatic status can release iron and ferritin from damaged hepatocytes, a process sustained also by the concomitant high serum levels of ALT. Furthermore, iron accumulation in CHC (11), causes liver damage due to the oxidative stress which increases necrosis/apoptosis of hepatocytes, activation of hepatic stellate cells, and fibrogenesis through the proliferation of actin and collagen (12, 13).

Several studies (14-17) reported that patients with CHC present mild to moderate hepatic iron accumulation, which significantly worsens clinical outcomes, leading to an increased risk of hepatocellular carcinoma. We obtained statistically significant differences for ALT, AST, serum iron, TIBC, ferritin between the two groups of mild and severe fibrosis. There is growing evidence that even mildly increased amounts of iron in the liver can be damaging, particularly if combined with other hepatotoxic factors (such as chronic viral hepatitis). After we excluded the values of transaminases, age and gender to avoid confounders influence, using multiple linear regression analysis, only ferritin and transferrin levels were the independent predictors for advanced liver fibrosis, while transferrin and serum iron levels were the independent predictors for severe necroinflammatory activity. These results are in accordance with several other reports (15, 18), suggesting that serum iron markers can represent surrogate markers for the severity of liver disease; still, these observations should be carefully interpreted, and the level of serum iron markers should be monitored in dynamics, as other interferences cannot be excluded. The interaction between hepcidin (the main regulator of iron homeostasis via the interleukin-6 (IL-6)/STAT3 pathway)and ferroportin (the trans membrane iron transporter) plays crucial roles through down-regulation of iron release from enterocytes and phagocytes; furthermore, mutations in several iron-metabolism related genes may also lead to iron alterations (19). Recent in vitro studies on molecular mechanisms of iron intervention on HCV life cycle generated divergent results, since iron promotes HCV translation (via internal ribosome entry site IRES and translation initiation factor eIF 3), but also inhibits the viral RNA-dependent RNA polymerase NS5B (20). Nevertheless, we found no significant correlations between HCV viral load and iron metabolism markers.

Still, recent studies detected that serum iron markers, and in particular serum ferritin levels, were independently associated with poor response to standard of care therapy as the oxidative stress induced by iron blocks JAK-STAT pathway (16). In this study we noted that advanced liver fibrosis is present in a high percentage of patients, and it is independently associated with ferritin and transferrin levels, indicating that these can be predictive markers for a group of hard-to-treat patients with HCV infection. Considering the fact that most patients in Romania are infected with HCV genotype 1b, the post-therapeutically prognosis is furthermore considered unfavorable.

The weak point of this study was the small size of patients with HCV infection included in analysis, and the strong point was that data available on the HCV infection in Romania is scarce, and studies in this field can complete the overall epidemic picture.

Concluding, the present study revealed two aspects: (1) a part of naive CHC patients has blood serum iron markers within the normal range; however, the percentage of those with advanced liver fibrosis is high and is significantly correlated to serum levels of iron, ferritin and TIBC; (2) even though the iron markers do not differ significantly in patients with low or high HCV viremia, serum ferritin and transferrin levels seem to play an important role to determine the severity of liver disease, related both to the liver fibrosis and necroinflammatory activity.
